# Curing Behavior and Thermomechanical Performance of Bioepoxy Resin Synthesized from Vanillyl Alcohol: Effects of the Curing Agent

**DOI:** 10.3390/polym13172891

**Published:** 2021-08-27

**Authors:** Zhenyu Wang, Pitchaimari Gnanasekar, Sandeep Sudhakaran Nair, Songlin Yi, Ning Yan

**Affiliations:** 1 Research Institute of Wood Industry, Chinese Academy of Forestry, Beijing 100091, China; wangzhenyu1992@126.com; 2 Beijing Key Laboratory of Wood Science and Engineering, Beijing Forestry University, Beijing 100083, China; 3 Department of Chemical Engineering and Applied Chemistry, University of Toronto, Toronto, ON M5S 3E5, Canada; mari.gnanasekar@utoronto.ca (P.G.); sandeepnair217@gmail.com (S.S.N.); 4 John H. Daniels Faculty of Architecture, Landscape, and Design, University of Toronto, Toronto, ON M5S 2J5, Canada

**Keywords:** bio–based epoxy resin, vanillyl alcohol, aliphatic amines, curing system, thermomechanical properties

## Abstract

In order to reduce the dependency of resin synthesis on petroleum resources, vanillyl alcohol which is a renewable material that can be produced from lignin has been used to synthesize bioepoxy resin. Although it has been widely reported that the curing reaction and properties of the cured epoxies can be greatly affected by the molecular structure of the curing agents, the exact influence remains unknown for bioepoxies. In this study, four aliphatic amines with different molecular structures and amine functionalities, namely triethylenetetramine (TETA), Tris(2-aminoethyl)amine (TREN), diethylenetriamine (DETA), and ethylenediamine (EDA), were used to cure the synthesized vanillyl alcohol–based bioepoxy resin (VE). The curing reaction of VE and the physicochemical properties, especially the thermomechanical performance of the cured bioepoxies with different amine functionalities, were systematically investigated and compared using different characterization methods, such as DSC, ATR–FTIR, TGA, DMA, and tensile testing, etc. Despite a higher curing temperature needed in the VE–TETA resin system, the cured VE–TETA epoxy showed a better chemical resistance, particularly acidic resistance, as well as a lower swelling ratio than the others. The higher thermal decomposition temperature, storage modulus, and relaxation temperature of VE–TETA epoxy indicated its superior thermal stability and thermomechanical properties. Moreover, the tensile strength of VE cured by TETA was 1.4~2.6 times higher than those of other curing systems. In conclusion, TETA was shown to be the optimum epoxy curing agent for vanillyl alcohol–based bioepoxy resin.

## 1. Introduction

Nowadays, epoxy resin has become an indispensable thermosetting resin for many industrial fields and can be widely used as high–performance coatings, adhesives, and composites, etc., because of its premium physicochemical properties and excellent compatibility with most materials [1]. However, current epoxy resin production is heavily dependent on petroleum–based resources and employs a great amount of reprotoxic bisphenol A which is used as a chemical precursor for more than 80% of the epoxy production worldwide [2,3]. Taking into account the decline in petroleum resources and the rise in environmental awareness, the existing mode of epoxy resin production is inconsistent with the concept of green and sustainable development. Therefore, there is a growing interest in utilizing biobased biomolecules (especially nonharmful monomers containing aromatic bifunctionalities) to produce epoxy polymers.

In recent years, bioepoxies synthesized from natural renewable resources, such as plant oils, rosins, lignin, and lignin derivatives, have been explored [4,5,6,7,8,9]. Among these biomolecules, lignin, which makes up around 30% of woody biomass, was mostly used as a low–grade fuel by the traditional pulp and paper–making industries. Meanwhile, lignin and lignin derivatives have been regarded as a highly promising raw material for epoxy synthesis, due not only to their wide varieties of sources and excellent commercialization promises, but also to their aromatic and rigid molecular structures which are conducive for obtaining high–performance epoxies [10,11,12,13]. However, the direct utilization of lignin in epoxy production is hindered by its large variability and complex chemical structures [14]. As a result, some well–defined and monoaromatic platform chemicals that can be derived from lignin, such as vanillin and vanillin derivatives, have gained significant attention. The interest in vanillin and vanillin derivatives is further fueled by the commercialization of the lignin–to–vanillin process [15,16,17,18]. Vanillyl alcohol is a main component of lignin–derived aromatic diols by the reduction of vanillin. Because of the presence of a difunctional hydroxyl group (−OH) in the vanillyl alcohol, it has been used as an attractive chemical platform for the synthesis of renewable epoxies. It has been reported that vanillyl alcohol can be converted into difunctional epoxy monomers by glycidylation and the resulting epoxy resins show satisfying performance and yields [11,19].

For epoxies, a curing agent is required to cure the thermoset resin and to build the desired cross–linking networks that determine the final properties of the cured resins. There are a variety of curing agents, including amines, amides, acid anhydrides, polyphenols, etc., that are commonly used [20]. Currently, amine–based curing agents, as one of the basic types of curing agents, have gained more popularity than other types of curing agents in many fields [21,22]. In particular, aliphatic amines possessing high reactivity and low viscosity at room temperature are widely used to cure epoxy resins [23]. During the formation of cross–linking networks in the curing reaction, a number of parameters, such as curing time, curing temperature, the structure of epoxy, and the curing agent, are known to play a crucial role [21,24,25,26]. In previous studies, the effects of curing conditions on the conventional epoxy systems have been well investigated [27,28,29,30,31]. Thus, there exists a significant body of knowledge concerning how different types of curing agents affect the structure and property relationship for conventional petroleum–derived epoxy resins. These fundamental understandings contribute greatly to the optimization of epoxy resin formulations targeting specific applications. However, no systematic study has looked into the influence of the molecular structure of the curing agent on the curing behavior and performance of the vanillyl alcohol–based bioepoxy resins. Vanillyl alcohol–based bioepoxies have different molecular structures from the conventional petroleum–based bisphenol A type of epoxy resins, and the crosslinker plays an important role in determining the cross–linking structure of the cured resin. In order to better design these bioepoxy systems for broad adoption by the industry, a thorough understanding of how the structure–property relationship of this type of epoxy resin depends on the structure of the crosslinker is highly necessary.

Therefore, in this study, four common aliphatic amines with a varied quantity of amino groups and different molecular structures (linear vs. branched) were chosen as curing agents to react with a bioepoxy resin synthesized from vanillyl alcohol. The curing agents included triethylenetetramine (TETA), Tris(2-aminoethyl)amine (TREN), diethylenetriamine (DETA), and ethylenediamine (EDA). The comprehensive characteristics of the bioepoxy resin systems, such as curing behavior, chemical structure, chemical resistance, thermal stability, thermomechanical properties, tensile performance, etc., were examined respectively using various analysis techniques including differential scanning calorimetry (DSC), attenuated total reflectance–Fourier transform infrared spectroscopy (ATR–FTIR), thermogravimetric analysis (TGA), dynamic mechanical analysis (DMA), and universal mechanical tester. By investigating the effect of the curing agent on the physicochemical properties of the cured vanillyl alcohol–based bioepoxies, the main focus of the present work is to elucidate the interactions between the bioepoxy and the curing agent to aid the optimization of the bioepoxy systems to promote industrial applications of these novel bioepoxy resins.

## 2. Materials and Methods

### 2.1. Materials

**Figure 1:**
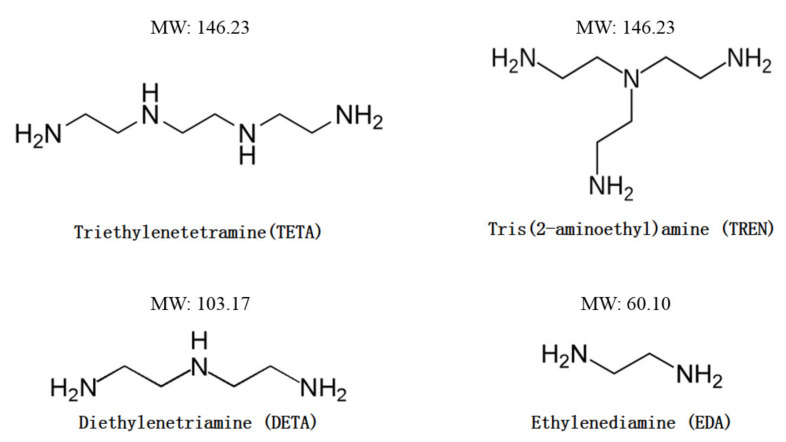
Structures of four different aliphatic amine curing agents used in this study.

The chemicals for synthesizing and curing the vanillyl alcohol–based bioepoxy resin (VE) were supplied by Sigma Aldrich or Fisher Chemical. Benzyltriethylammonium chloride (TEBAC, 99%) was selected as the phase transfer catalyst in the etherification reaction between vanillyl alcohol (98%) and epichlorohydrin (99%). The sodium hydroxide (99.1%) was used to initiate the ring–closing reaction of the chlorohydrin ether intermediate to reform oxirane rings in the resulting bioepoxy resin. The structure and molecular weight of the curing agents, namely TETA (97%), TREN (96%), DETA (99%), and EDA (99%), are presented in Figure 1. All the reagents mentioned above were used as received.

### 2.2. Synthesis of Vanillyl Alcohol–Based Bioepoxy Resin

**Figure 2:**
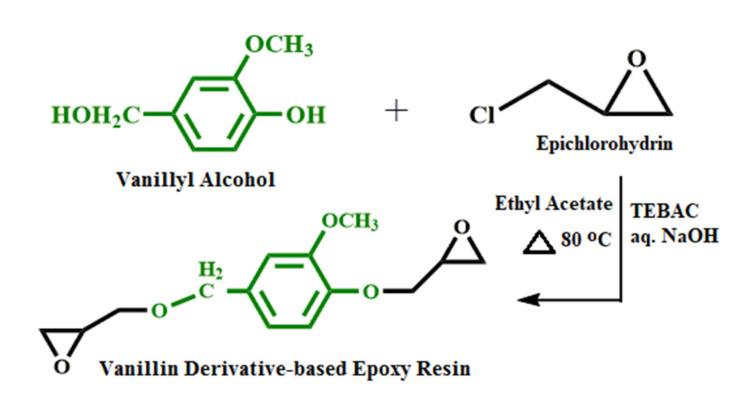
Reaction scheme of vanillyl alcohol–based bioepoxy resin synthesis.

The synthesis process of vanillyl alcohol–based bioepoxy resin was carried out by the following procedure. A three–neck round bottom flask equipped with a mechanical stirrer was charged with vanillyl alcohol, TEBAC, and epichlorohydrin in the molar ratio of 1: 1: 10. After 1 h mechanical stirring at room temperature to assure the complete dissolution of reactants, the mixture was gradually heated up to 80 °C and was left under stirring for 0.5 h. Then, the flask was cooled down to room temperature again. For each mole of vanillyl alcohol involved in the reaction system, 0.1 mol TEBAC and 5 mol sodium hydroxide (800 mL aq) were added into the flask, and left to constant stirring for 0.5 h. Afterwards, the ethyl acetate and distilled water were added into the resulting solution in the volume ratio of 1:1. Subsequent to another stirring for several minutes, the two–phase mixture was left to stand for 1 h to separate into two layers. After the completion of separation, the top organic phase layer containing bioepoxy resin was collected and then washed with distilled water. Magnesium sulfate, anhydrous, was used to dry the organic layer, and the precipitate was filtered. The final bioepoxy resin was obtained by evaporating the filtrate on a rotary evaporator to remove the ethyl acetate as well as excess epichlorohydrin in the organic layer. The synthesis approach of the vanillyl alcohol–based bioepoxy resin is shown in Figure 2. The structural information of the synthesized bioepoxy resin has been provided in our previous study [32].

### 2.3. Curing Process of the Bioepoxy Resin

Four aliphatic amines with different structures and functionalities were selected to cure the vanillyl alcohol–based bioepoxy resin. Owing to each of the active hydrogens in aliphatic amines supposedly only consuming one epoxide group, the curing agents were added into VE stoichiometrically, and the weight ratios of VE to each curing agent are shown in Table 1. The samples cured with different curing agents were labelled as VE–TETA, VE–TREN, VE–DETA, and VE–EDA, respectively. In order to decrease the viscosity of VE and achieve its full mixing with the curing agents, acetone of the same weight as VE was used as a diluent. After mixing, the mixture of VE and curing agent was magnetically stirred for 4 h, and then the acetone was evaporated by standing the mixture in a fume hood overnight. A drying oven was used to accomplish the curing process as per the following schedule: 0.5 h −60 °C, 0.5 h −80 °C, 0.5 h −100 °C, 0.5 h −135 °C, and 1 h −180 °C. Prior to further testing, the solid bioepoxies were molded into the required shape and size according to the experimental methods described here.

### 2.4. DSC Analysis

The curing process of the vanillyl alcohol–based bioepoxy resin with different curing agents was recorded on a Q100 DSC (TA Instruments, USA) analyzer. A high–volume pan was used to seal around 7 mg of VE–curing agent mixtures in the measurement. The experiment was carried out under a nitrogen atmosphere, and the curing temperature was increased from 25 to 250 °C at a heating rate of 10 °C /min.

### 2.5. ATR–FTIR Analysis

A Nicolet iS50 FTIR (Thermo Fisher Scientific, USA) spectrometer was applied to identify the chemical structure of vanillyl alcohol–based bioepoxy resin cured with different curing agents. Slices of different cured samples with the dimension of nearly 10 mm × 10 mm × 0.6 mm were collected to carry out the ATR–FTIR analysis. The spectrum of each curing system was measured in the wavenumber range of 4000 to 400 cm^−1^ with 64 scans and a resolution of 4 cm^−1^.

### 2.6. Swelling and Chemical Resistance Testing

The swelling and chemical resistance studies of different bioepoxy systems were performed on small pieces of cured resin with the same dimension of nearly 10 mm × 10 mm × 0.6 mm. Samples were immersed in sealed vials containing 10 mL of different solvents for 168 h at room temperature. Toluene was used in the testing of swelling ratio and gel content (resistance to organic solvent) [33]. NaOH (3 M) and HCl (3 M) aqueous solution were used to examine the samples’ resistance to alkaline and acid medium, respectively. The weights of the dried samples before and after immersion were measured to calculate the mass changes. The samples in the swelling tests were weighed every 24 h to obtain the plots for the swelling ratios. All tests were conducted in triplicate to determine a mean mass change.

### 2.7. Thermogravimetric Analysis

The thermal decomposition behavior of all cured VE samples was investigated with TGA. The measurement was carried out using a Q500 (TA Instruments, USA) thermogravimetric analyzer. Small pieces of the samples of around 10 mg in all experiments were placed in a crucible with a dynamic scan to obtain the thermogravimetric profiles. The temperature increased from room temperature to 600 °C at the heating rate of 10 °C/min with a 60 mL/min nitrogen flow.

### 2.8. Dynamic Mechanical Analysis

The Q800 dynamic mechanical analyzer (TA Instruments, USA) was applied to study thermomechanical responses of the cured resins by operating in a multi–frequency–strain mode. The measurement frequency and oscillation amplitude were set to 1 Hz and 15 μm, respectively. Before the measurement, different cured resin samples were cut into rectangular bars with a dimension of approximately 15 mm × 4 mm × 0.6 mm, and fixed on the clamp. With a heating rate of 3 °C/min, the storage modulus and loss tangent were collected from room temperature to 180 °C.

### 2.9. Tensile Testing

The tensile testing of the cured resin samples was conducted on an Instron 3367 universal testing machine equipped with a 2 kN load cell. After being machined into dog–bone geometry according to ASTM D 638–Type V specifications (63.50 mm in length and 9.53 mm in width), the specimens were mounted on the tester by the clamping jaws with a span of 25 mm. The crosshead speed in testing was set to 10 mm/min^−1^. Five specimens of each VE–curing agent system were measured to obtain the average value.

## 3. Results and Discussion

### 3.1. Curing Behavior of the Synthesized Bioepoxies

**Figure 3:**
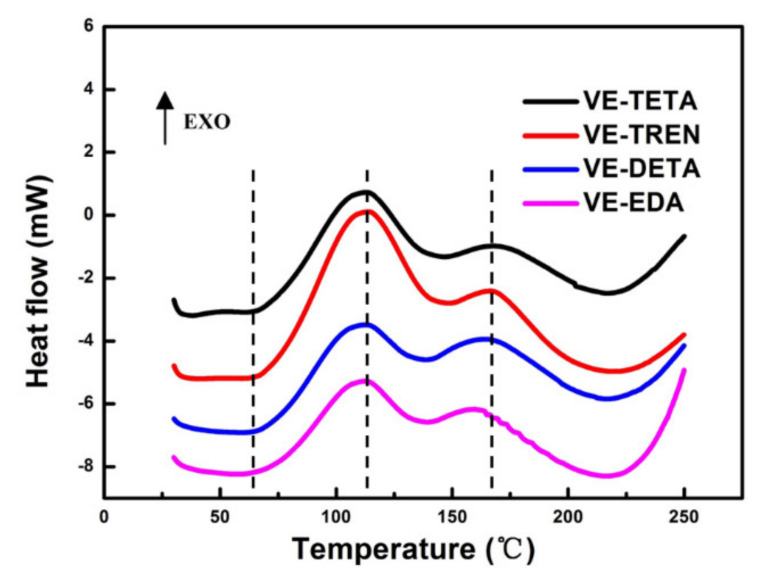
DSC curves of vanillyl alcohol–based epoxy with various curing agents.

In Figure 3, the DSC curves of the heat–flow data versus temperature were plotted to show the curing process of the vanillyl alcohol–based bioepoxy resin with different curing agents. From the two obvious peaks appearing in the curves of every sample, the different polyaddition stages in the curing process can be observed, which were possibly attributable to the reaction between the epoxy group with primary amines and secondary amines. It has been reported that the two types of amine groups coexist during the curing process but differ in reactivity. Primary amines are easier and faster to react than secondary amines [34,35,36]. In this study, all the reactants were stoichiometrically mixed with each other, thus every hydrogen atom in the two different amine groups was expected to react with one epoxide group. In theory, the reaction of partial hydrogen atoms linked to primary amine would first occur and also form more secondary amines, then the original and/or produced secondary amines would further react until all epoxy groups were exhausted. Consequently, the first peaks of DSC curves at low temperature were mainly corresponding to the reaction between epoxy and highly reactive primary amines, while the second ones were related to the reaction of secondary amines. Furthermore, compared with the second peak at a higher temperature in the same DCS curve, the first peak usually exhibited a higher reaction heat, which indicates a higher reaction intensity in the early curing stage. This may be also the result of the higher reactivity of primary amines, as well as the lower steric and diffusional restrictions before the completion of a relatively tight cross–linking in the second curing stage.

According to the characteristics of the DSC thermogram in Figure 3, some important points in the curing process, including where the reaction began, peaked, and finished, were determined. The corresponding temperatures were represented by T_i_, T_p_, and T_f_, respectively, in Table 2. Among the samples cured by the four different amines, the two–stage curing process was not significantly affected by the type of curing agents. However, VE–EDA showed lower initial and peak temperatures than the others. This probably resulted from the higher proportion of primary amines in EDA and the lower molecular weight of EDA, which were both in favor of the reactions occurring at relatively low temperatures [36,37]. This trend was more obvious in the second peak, which also indicated the small molecules of EDA were easier to move and react with VE when parts of the cross–linking network had started to form. In contrast, with the increase of the molecular weight, the T_i_ and T_p_ in VE–TETA and VE–TREN rose remarkably, suggesting a higher energy consumption than VE–EDA during the curing process.

### 3.2. Chemical Structure and Chemical Resistance of the Cured Bioepoxies

**Figure 4:**
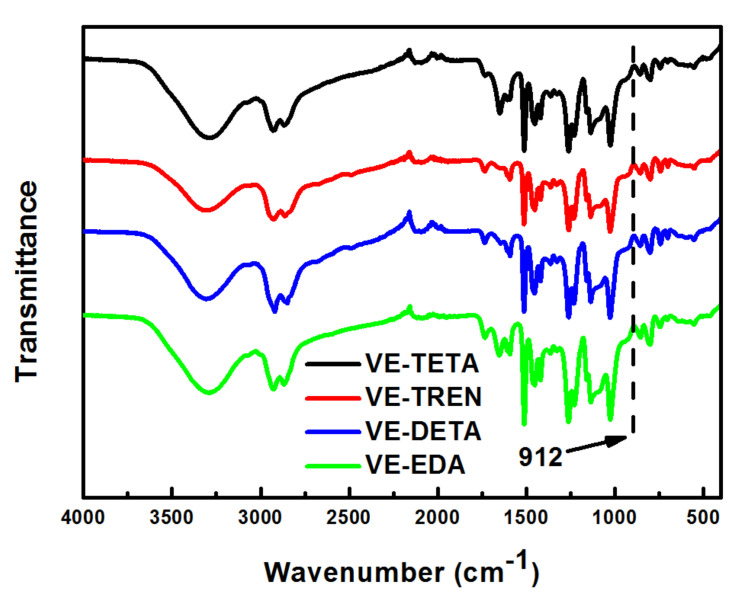
ATR–FTIR analysis of vanillyl alcohol–based bioepoxy resin cured by various curing agents.

From the ATR–FTIR tests of the cured bioepoxies, the chemical structure of the different VE–curing agent systems was obtained in Figure 4. All cured resins with different curing agents showed a similar spectrum, mainly because all of them were cured by aliphatic amines and the discrepancies in functional groups were limited. It is noteworthy that, after curing, no characteristic peak of epoxide group was found at the wavenumber of 912 cm^−1^, which confirmed that most of the oxirane rings were depleted by the curing reaction [38]. However, considering the stoichiometric amount of reactants used in the curing process, it is hard to guarantee the full contact of every epoxy group with active hydrogens in amines as a result of the steric and diffusional restrictions. Consequently, apart from the reaction between bioepoxies and curing agents, as the curing temperature increased up to 180 °C, it was likely that some epoxide groups were consumed by the hydroxyl–epoxide reaction and/or epoxy homopolymerization [39].

**Figure 5:**
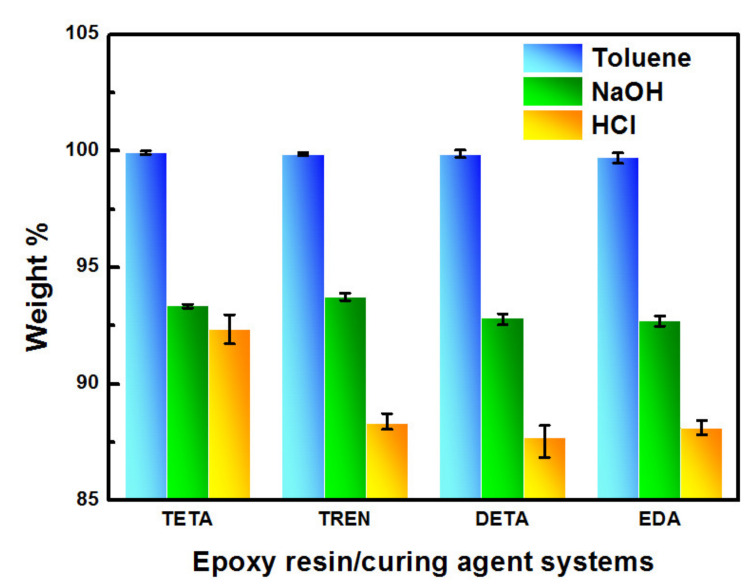
Gel content and acidic and alkaline resistance of the cured bioepoxies with various curing agents.

Additionally, the gel content of the cured bioepoxy resin was also detected to evaluate the degree of the curing process and the resistance of the cured resin to organic solvents. As shown in Figure 5, the gel contents of all cured samples in toluene were more than 99%, indicating that the reactants in the curing process were nearly completely incorporated into the polymer networks and the curing degree of the samples cured with different curing agents was almost the same [40]. Moreover, the resistance of the cured resins to the acidic and alkaline medium was examined. The results are also presented in Figure 5. Immersed in NaOH (3 M) and HCl (3 M) solutions for 168 h, vanillyl alcohol–based bioepoxy resin showed a better resistance to alkaline medium than acidic medium. All the cured systems had a similar weight reduction magnitude of around 7% in NaOH solution. Although there were some changes in the remaining weight between the different curing systems, the differences were small and insignificant. In HCl solution, the weight loss of the cured resins surged to near 13%, except for the VE–TETA samples which exhibited a much higher remaining weight (92.35%) than the others. The better chemical resistance in VE–TETA may be related to a higher cross–linked network and the structural characteristics of TETA [41,42].

**Figure 6:**
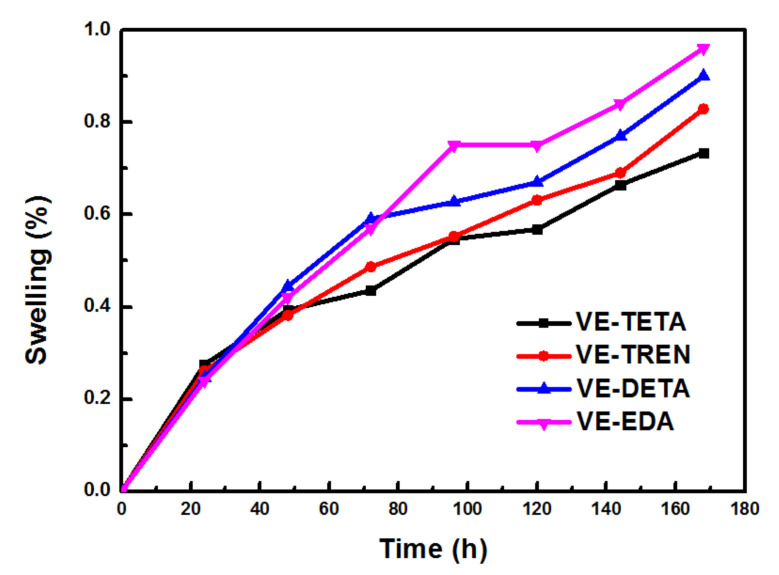
Swelling behaviors of the different vanillyl alcohol–based bioepoxy resin systems.

In addition to organic, acidic, and alkaline resistance, the swelling ratios of the cured epoxies were determined as a function of immersion time in toluene in Figure 6. Initially (before 24 h), all the samples showed a relatively quick increase in swelling, as a result of the penetration of the solvent into structural defects and cavities on the surface of the samples [42]. Then, a slower and continuous growth of swelling was observed in each sample, indicating the gradual immersion of toluene into the molecule intervals in the network structures. When comparing the differences between the different curing agent systems, after 168 h immersion, the VE–EDA sample showed the highest swelling ratio, while the lowest value could be found for the VE–TETA sample, probably because of a more stable network structure with a stronger crosslinking in the VE–TETA sample.

### 3.3. Thermal Properties of the Cured Bioepoxies

**Figure 7:**
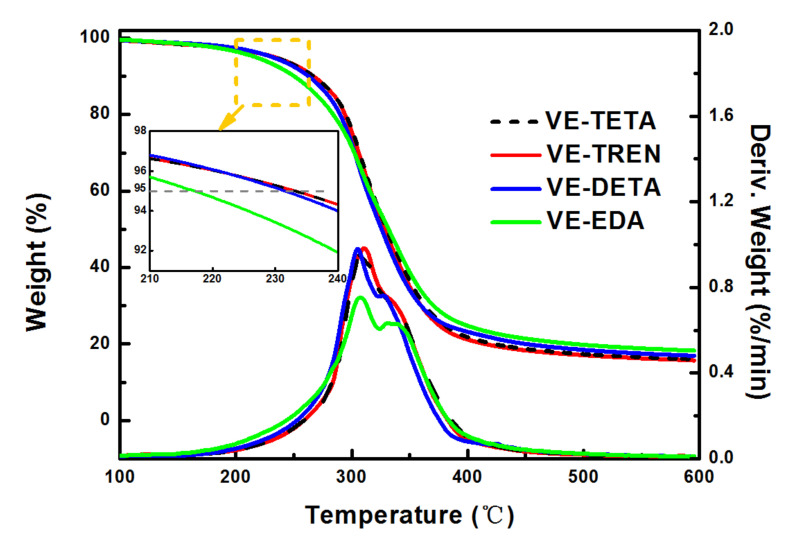
TGA curves of vanillyl alcohol–based bioepoxy resin cured by various curing agents.

The thermal property testing of the resins cured with different curing agents was conducted by TGA. On the basis of the thermogravimetric (TG) and differential thermogravimetric (DTG) curves shown in Figure 7, the main degradation stage of all samples was concentrated in the 200~400 °C temperature range, and the weight loss rate peaks were generally observed at around 310 °C, accompanied by a high–temperature shoulder peak. As reported by the study of Wu et al., this result of two weight–loss peaks in the DTG curves was largely caused by the rupture of different segments in the polymer networks [43]. The degradation temperatures at 5% (T_5%_), 10% (T_10%_), and 50% (T_50%_) weight loss are depicted in Table 3, and T_p_ represents the temperature at which the weight loss rate reached the maximum. Comparing the systems cured with different curing agents, the degradation of VE–EDA illustrated the lowest onset temperature (217.17 °C), indicating a relatively low thermal stability [44]. With the increase of amine groups and active hydrogens in curing agents, the thermal degradation onset temperature of the cured bioepoxies rose gradually, reaching 231.59 °C for VE–DETA, 233.16 °C and 233.24 °C for VE–TETA and VE–TREN, respectively. The rise in initial degradation temperature was mainly due to the formation of tighter cross–linking networks in the cured resin [45]. Interestingly, the final residue value at 600 °C showed the opposite results to the onset temperature, which was similar to what was reported by Gowda and Mahendra [46]. As the temperature increased during the thermal degradation process, the cross–linked network disintegrated severely, while the basic structure of the polymers played a crucial role in preventing further weight loss, especially at high temperatures. Thus, the higher percentage of thermally stable aromatic structure in the VE–EDA system than others might be the reason for the obvious degradation rate decline in the DTG curves and the highest residue content at 600 °C in Table 3 [47,48]. However, it is worth noting that VE–TETA and VE–TREN were found to have better thermal properties since they showed a relatively higher degradation temperature in almost every stage of the degradation process among all the curing systems.

### 3.4. Dynamic Mechanical Analysis of the Cured Bioepoxies

**Figure 8:**
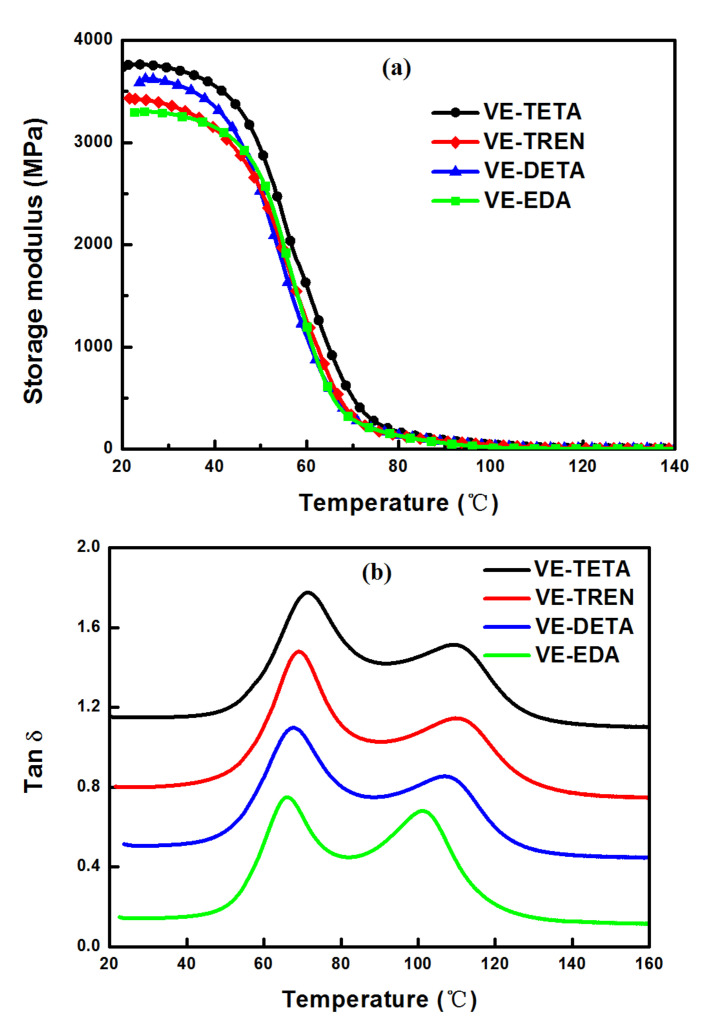
Storage modulus (**a**) and tan δ (**b**) curves of cured bioepoxies with different curing agents.

The thermomechanical features of the cured bioepoxy networks with different curing agents were determined by DMA. In Figure 8a,b, the storage modulus (E′) and loss factors (tan δ) are respectively plotted versus temperature. Correspondingly, the values relevant to the thermomechanical properties of the cured bioepoxies are presented in Table 4. The E′ represents the rigidity of the material, which was the highest for VE–TETA (3762.48 MPa) and the lowest for VE–EDA (3303.52 MPa). This was most likely attributed to the higher cross–linking density in VE–TETA networks than that in VE–EDA [45,49]. However, the E′ of VE–TREN was also significantly lower than that of VE–TETA, which seemed to be inconsistent with what was found for conventional epoxy resin systems [23]. This phenomenon was probably associated with the side chain of TREN molecules and the damping of the cured epoxy in subglass relaxation.

**Figure 9:**
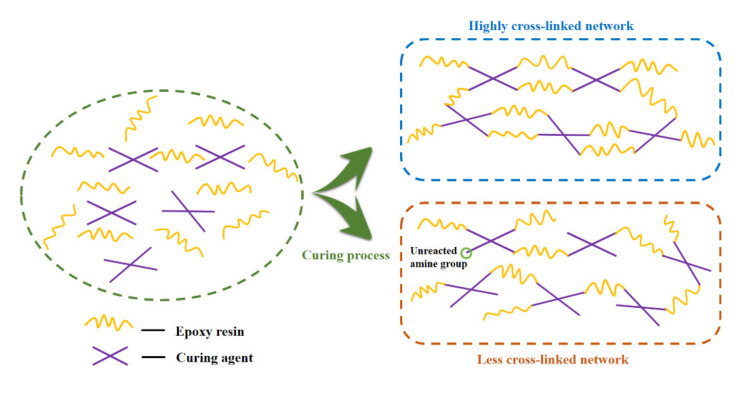
Schematic of the two types of crosslinking systems in the cured vanillyl alcohol–based bioepoxies.

In Figure 8b, the temperatures corresponding to tan δ peaks were used to determine the relaxation temperatures (T_α_). For all bioepoxy samples, there were two peaks found at around 70 °C and 105 °C, respectively in the tan δ curves, which indicated a heterogeneous polymer network [50,51]. This could be explained by two different regions present in the cured resins, namely the tighter regions with a high cross–linking degree as well as the looser regions with a relatively low cross–linking degree [52]. As discussed in the DSC section, the curing process of all VE–curing agent systems included two stages. During the first stage, highly reactive primary amines took the lead in reacting with epoxide groups, and a looser cross–linking network was built, owing to the incomplete reaction of the curing agents. Since the molecules were much easier to move in loose networks with fewer constraints, the crosslinks formed in this stage were capable of dissipating more energy by distortion [12,23]. Thus, in Figure 8b, the characteristic peak with low T_α_ corresponded to the looser regions in the whole cross–linked networks and displayed a high tan δ. As for the latter stage, more secondary amines, which had been reported to construct a stronger crosslinked network than the primary amines, started to be involved in the curing reaction [36]. The tight regions cured in this period were mainly reflected by the second peak in the tan δ curves. In the tight regions, stronger covalent bonds inside the polymer chains could be formed to prevent molecules from moving, thus the second peak displayed a low tan δ at high temperatures [53]. However, although the total amount of secondary amines in the whole curing process prevailed over that of primary amines, the large–scale molecular motions of the cured resin and the drastic decrease of E′ occurred at the same temperature as the first tan δ peak, implying the loose networks seemed to be the dominant structure in the cured systems. This might result from the steric restriction caused by the already formed networks during the curing process, which retarded the full crosslinking of tight networks and led to a high percentage of the loose networks in the entire cured system. The possible schematic representation of the two types of crosslinking systems is shown in Figure 9. When the tan δ curves of the resins cured by different amines were compared with each other, the VE–TETA and VE–TREN samples showed a relatively high T_α_ in both of the tan δ peaks, indicating a better thermomechanical property of the highly cross–linked VE–curing agent systems. Instead, the VE–EDA sample exhibited a lower T_α_ with a higher tan δ, which was consistent with the results mentioned in other sections that the VE–EDA cross–linking networks were comparatively weaker than the others.

### 3.5. Mechanical Properties of the Cured Bioepoxies

The mechanical performances of vanillyl alcohol–based bioepoxy resins cured by different curing agents are given in Table 5. The tensile strength reached the highest value in VE–TETA (32.94 MPa) and was considerably reduced in the other VE–curing agent systems. The order for tensile strength values was as follows: VE–TETA > VE–TREN > VE–DETA > VE–EDA. Evidently, with the increase in the functionality of the curing agent, a higher mechanical property was observed due to the formation of stronger cross–linking networks. Comparing bioepoxies cured by tetrafunctional TETA and bifunctional EDA, the tensile strength of TETA was more than twice that of EDA. Meanwhile, the influence of the molecular structure of the curing agent on the cured bioepoxy mechanical properties can be clearly revealed from the difference between the VE–TREN and VE–TETA samples. Although having a comparable cross–linking degree with VE–TETA, VE–TREN showed a much lower strength (72.25% of VE–TREN) and modulus (61.13% of VE–TREN) in tensile testing, which was similar to what was observed from the DMA test results. Likewise, this was also related to the branched structure of TREN. It was because the side chains increased the steric hindrance to negatively affect mechanical properties by disturbing the orientation of the molecule chains and causing a larger local stress [54]. These results illustrated the complex interplay between the type and number of the amine functionality and the molecular structure of the crosslinker in determining the final mechanical properties of the cured resin systems.

## 4. Conclusions

In this work, renewable vanillyl alcohol was used to synthesize vanillyl alcohol–based bioepoxy resin (VE) without bisphenol A. In order to systematically explore the effects of different curing agents on the bioepoxy curing characteristics and properties of the cured resins, four aliphatic amines with different structures and amine functionalities, including TETA, TREN, DETA, and EDA, were used in the curing process. According to the DSC measurements, chemical resistance and swelling tests, TGA and DMA analysis, and tensile tests, different curing agents had a significant influence on the curing behavior and properties of the cured bioepoxy resins. Among the four curing agents used in this study, TETA was the optimum crosslinker. Although the temperature required for curing was lowest for VE–EDA and rose with the increase in molecular weight of the curing agent, the cured VE–TETA sample exhibited a better overall performance than the other epoxy resin systems, including higher chemical resistance, thermal stability, tensile strength, modulus, and a lower swelling ratio. By analyzing the two–stage resin curing process and comparing the differences of performances between the various curing systems, the superior performance of the VE–TETA epoxy system mainly resulted from the stronger cross–linking networks formed in the curing reaction and the molecular structural characteristics of TETA itself. Since vanillyl alcohol–based bioepoxy has great potential to be used as a substitute for current petroleum–based thermosetting epoxy resins, especially bisphenol A–type epoxy resins, a better fundamental understanding of the epoxy resin and curing agent interactions will help to promote the industrial utilization of these bioepoxies to achieve a higher level of sustainability.

## Figures and Tables

**Table 1:** Weight ratios of vanillyl alcohol–based epoxy resin to curing agents for different curing systems.

Curing System	VE/Curing Agent Ratio
VE–TETA	100.00/18.33
VE–TREN	100.00/18.33
VE–DETA	100.00/15.51
VE–EDA	100.00/11.30

**Table 2:** Characteristic temperatures from DSC studies of the curing reactions.

	T_i_ (°C)	T_p1_ (°C)	T_p2_ (°C)	T_f_ (°C)
VE–TETA	64.42	112.71	167.30	214.60
VE–TREN	64.89	113.75	167.84	214.58
VE–DETA	61.81	112.18	165.20	214.04
VE–EDA	60.75	110.58	161.05	212.98

**Table 3:** Thermal properties of vanillyl alcohol–based bioepoxy resin cured by various curing agents.

	T_5%_ (°C)	T_10%_ (°C)	T_50%_ (°C)	T_p_ (°C)	Final Residue (%) at 600 °C
VE–TETA	233.16	267.05	330.17	309.13	16.04
VE–TREN	233.24	267.05	328.38	310.45	15.73
VE–DETA	231.59	262.66	325.80	305.06	16.95
VE–EDA	217.17	250.27	331.76	307.47	18.29

**Table 4:** Thermomechanical properties of cured bioepoxies.

	E′ (MPa)	T_α1_ (°C)	tan δ_1_	T_α2_ (°C)	tan δ_2_
VE–TETA	3762.48	71.37	0.69	109.21	0.43
VE–TREN	3425.32	69.07	0.75	110.49	0.42
VE–DETA	3619.76	67.63	0.66	107.12	0.42
VE–EDA	3303.52	66.11	0.67	101.07	0.60

**Table 5:** Mechanical properties of different bioepoxy systems.

	Tensile Strength (MPa)	Tensile Modulus (GPa)	Strain at Break (%)
VE–TETA	32.94 (±3.96)	2.47 (±0.30)	1.33 (±0.00)
VE–TREN	23.80(±3.83)	1.51(±0.67)	1.67(±0.47)
VE–DETA	18.92(±1.89)	2.84 (±0.28)	0.99 (±0.47)
VE–EDA	12.62 (±2.28)	0.58 (±0.10)	2.00 (±0.00)

## Data Availability

Data available on request.
